# Differential Proteome Analysis of a Flor Yeast Strain under Biofilm Formation

**DOI:** 10.3390/ijms18040720

**Published:** 2017-03-28

**Authors:** Jaime Moreno-García, Juan Carlos Mauricio, Juan Moreno, Teresa García-Martínez

**Affiliations:** 1 Department of Microbiology, Agrifood Campus of International Excellence ceiA3, University of Cordoba, 14014-Cordoba, Spain; b62mogaj@uco.es (J.M.-G.); mi1gamaj@uco.es (J.C.M.); 2 Department of Agricultural Chemistry, Agrifood Campus of International Excellence ceiA3, University of Cordoba, 14014-Cordoba, Spain; qe1movij@uco.es

**Keywords:** *Saccharomyces cerevisiae*, flor velum, GO Terms, OFFGEL electrophoresis, LTQ Orbitrap XL MS

## Abstract

Several *Saccharomyces cerevisiae* strains (flor yeasts) form a biofilm (flor velum) on the surface of Sherry wines after fermentation, when glucose is depleted. This flor velum is fundamental to biological aging of these particular wines. In this study, we identify abundant proteins in the formation of the biofilm of an industrial flor yeast strain. A database search to enrich flor yeast “biological process” and “cellular component” according to Gene Ontology Terminology (GO Terms) and, “pathways” was carried out. The most abundant proteins detected were largely involved in respiration, translation, stress damage prevention and repair, amino acid metabolism (glycine, isoleucine, leucine and arginine), glycolysis/gluconeogenesis and biosynthesis of vitamin B9 (folate). These proteins were located in cellular components as in the peroxisome, mitochondria, vacuole, cell wall and extracellular region; being these two last directly related with the flor formation. Proteins like Bgl2p, Gcv3p, Hyp2p, Mdh1p, Suc2p and Ygp1p were quantified in very high levels. This study reveals some expected processes and provides new and important information for the design of conditions and genetic constructions of flor yeasts for improving the cellular survival and, thus, to optimize biological aging of Sherry wine production.

## 1. Introduction

*Saccharomyces cerevisiae* flor yeast strains are very interesting for winemaking purposes due to their influence on the sensory properties of Sherry type wines [1,2]. These wines are produced from a process named “biological aging”, a process conducted in many regions around the world, including Spain, France, Italy, South Africa, Hungary, Armenia, USA (California) and Southern Australia. In Southern Spain, wine is biologically aged in a so-called “criaderas system”, which is regarded as the greatest contribution of this region to winemaking worldwide.

During biological aging, flor yeasts develop an oxidative metabolism (respiration) under harsh survival conditions, including high ethanol and acetaldehyde concentrations, oxidative stress and low pH. Buoyancy and spontaneous, natural immobilization by formation of a biofilm (flor velum) on the surface of wine provide an effective mechanism that protects the cells and facilitates survival and proliferation under the adverse conditions during the biological aging. In fact, the formation of a biofilm at the wine-air interface brings yeasts into contact with an oxygen-rich zone where they can efficiently metabolize ethanol and glycerol. Yeast proliferation under the prevailing oxidative conditions of biological aging is also facilitated by the antioxidant defense system protecting cells from reactive oxygen species (ROS) formed during the oxidative metabolism of non-fermentable carbon sources (ethanol and glycerol) [3,4].

The ability of flor yeasts to float on the surface of wine was initially ascribed to a high surface hydrophobicity of yeast cells [5] resulting from a specific composition of their walls. Ishigami et al. [6] and Fidalgo et al. [7] among other authors have shown the cell wall protein Flo11p to be involved in the formation of yeast biofilms. The process also involves other proteins, such as Hsp12p, Nrg1p and Btn2p [6,8,9]. Organelles and cellular components, such as cell wall and mitochondria, play prominent roles in the oxidative metabolism of flor yeasts and hence in their antioxidant defense mechanisms [4].

This study is part of a sequence of proteomic research that was focused on specific flor yeast organelles/processes [4,10,11,12]. Distinctively, a broad vision of the flor yeast proteome is provided, and those organelles, biological processes and pathways with more proteins and in higher contents are highlighted in this work. We carried out a proteomic technique, using an OFFGEL fractionator coupled to an LTQ Orbitrap XL MS, to detect as many proteins as possible in a flor yeast at the initial stages of biofilm formation in a standard medium under biofilm formation condition (BFC). As a reference, the non-biofilm formation condition (NBFC), where ethanol and glycerol were replaced by glucose, was used [13]. Proteins were classified as “biological process” and “cellular component” GO or Gene Ontology (defines concepts/classes used to describe gene function and relationships between these concepts) Terms, and pathways according to the SGD (*Saccharomyces* Genome Database) and UniProt databases in order to elucidate the overall proteome of the flor yeast strain. Lastly, the GO Term finder tool of SGD and a GO Term or pathway ratio were utilized to identify the GO Terms, pathways and particular proteins exhibiting the greatest differences between the two tested conditions.

## 2. Results and Discussion

A total of 413 proteins was detected under the BFC and 611 under NBFC, of which 266 were more abundant under BFC (viz., 199 proteins specifically detected under BFC plus 67 present under both conditions, but in amounts more than two-times greater under BFC). On the other hand, 439 proteins were more abundant under NBFC (viz., 397 proteins detected only under this condition plus 42 with a higher content under it). A total of 105 proteins was found in similar amounts under BFC and NBFC. Average protein content in mol % was 0.24 in the case of BFC and 0.16 in NBFC.

**Figure 1:**
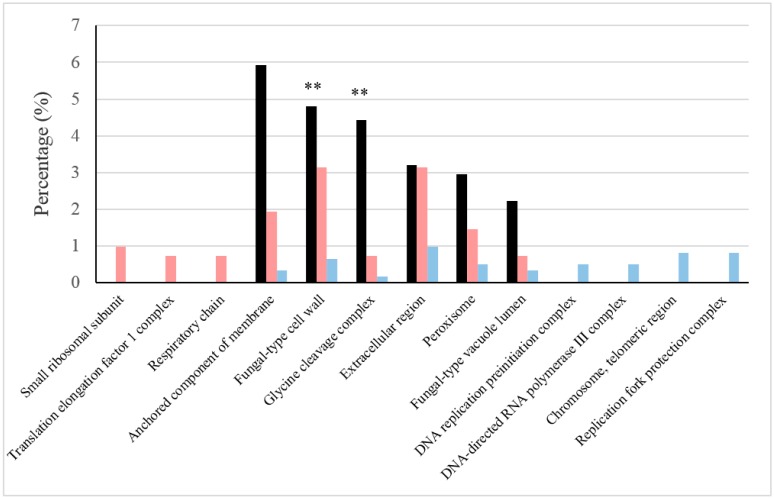
Relevant cellular components under BFC and NBFC. These comprise cellular components with high values of the GO (Gene Ontology) Term ratio (black) and those in which the frequencies of more abundant proteins were reported in one of the conditions, but not in the other (sides of the graphs). The frequency of proteins under BFC is shown in pink and that under NBFC in blue. GO Term *p*-values are represented with ** showing <0.05.

**Figure 2:**
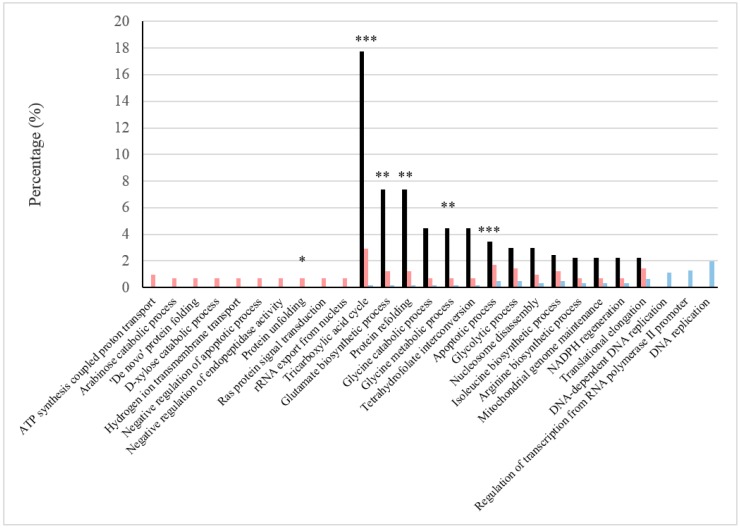
Relevant biological processes under BFC and NBFC. These comprise biological processes with high values of the GO Term ratio (black) and those in which the frequencies of more abundant proteins were reported in one of the conditions, but not in the other (sides of the graphs). The frequency of proteins under BFC is shown in pink and that under NBFC in blue. GO Term *p*-values are represented with *, ** and *** showing <0.1, <0.05 and <0.001, respectively.

**Figure 3:**
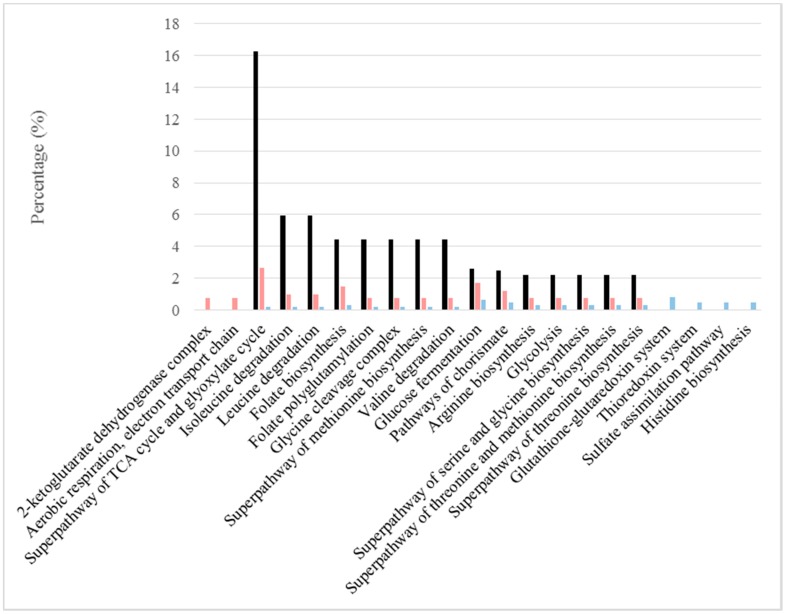
Relevant pathways under BFC and NBFC. These comprise pathways with high values of the GO pathway ratio (black) and those in which the frequencies of more abundant proteins were reported in one of the conditions, but not in the other (sides of the graphs). The frequency of proteins under BFC is shown in pink and that under NBFC in blue.

The roles of the most abundant proteins under each type of condition were elucidated from the GO Term or pathway ratios for cellular components, biological processes and pathways (see Figure 1, Figure 2 and Figure 3). Mutually related GO Terms and pathways (e.g., the respiratory chain and ATP synthesis, which are coupled to proton transport) were sorted into broader groups: cellular respiration, stress damage prevention and repair, protein biosynthesis, cell wall and extracellular region, peroxisome, vacuole, proteolysis regulation and apoptosis, catabolism of several sugars, amino acid metabolism, glycolysis/gluconeogenesis and biosynthesis of folate.

### 2.1. Cellular Respiration

**Figure 4:**
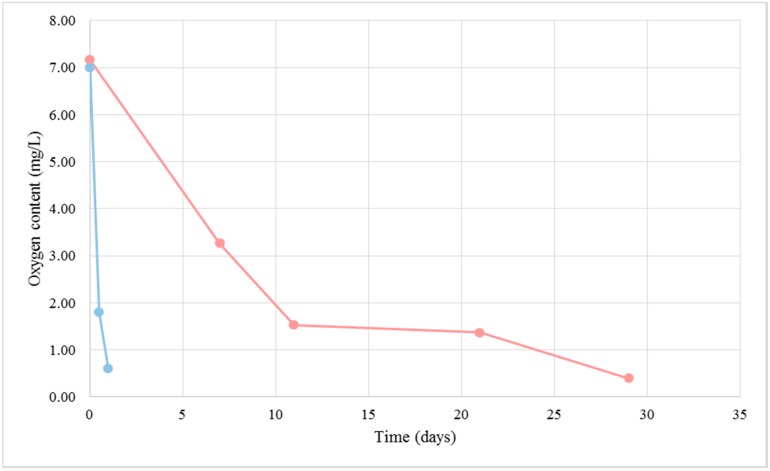
Dissolved oxygen content in the BFC medium (pink) and NBFC medium (blue).

**Figure 5:**
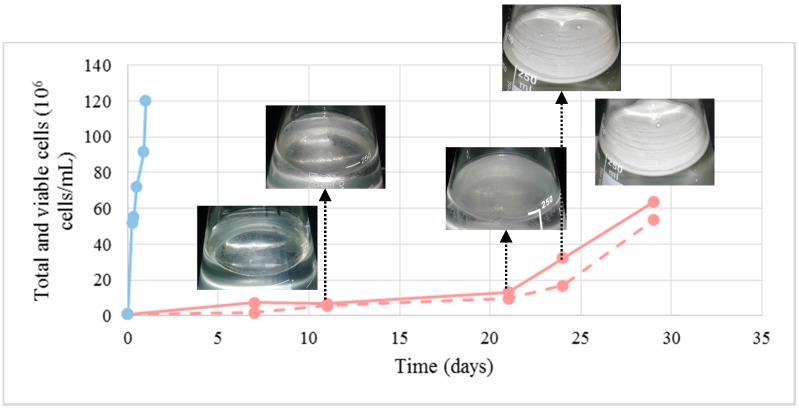
Total (continuous lines) and viable cells (dashed lines) numbers of yeast growing in the flor velum under BFC (pink) and in suspension in the medium under NBFC (blue). All yeast cells in the NBFC medium at the sampling time (12 h) were viable.

A large number of proteins present in cellular components, biological processes and pathways participating in cellular respiration metabolism were detected under BFC. Biofilm-forming flor yeasts are known to have an oxidative metabolism [14], also confirmed by the oxygen consumption observed in this study (Figure 4). In the same figure and Figure 5, it is possible to distinguish three different phases in the biofilm development: the first one about after the first 10 days when a rapid consumption of oxygen takes place in the culture medium, the second one when the consumption slows down and, finally, the last phase until the dissolved oxygen becomes exhausted. After about 30 days, the flor velum is fully formed under the liquid surface, and flor yeast cells quickly uptake the oxygen that is dissolving in this region (Figure 5). As expected, yeast cells from NBFC consumed the medium oxygen in the first 24 h. In this study, we identified two proteins taking part in the tricarboxylic acid cycle (TCA) under NBFC and twelve under BFC (*p*-value < 0.001 considering BFC more abundant proteins); one (Mdh1p) was present at 1.02 mol % under BFC, but absent under NBFC. This result is consistent with a balanced cellular redox process in flor yeasts [15], where Mdh1p acts as an NADH shuttle component. Two respiratory chain proteins were only detected under BFC (Cor1p, Cyb2p) and only one under the two conditions (Qcr6), at 0.22 mol % under BFC versus only 0.10 mol % under NBFC (Table 1 and the Supplementary Material). The target proteins taking part in hydrogen ion transmembrane transport and ATP synthesis coupled to proton transport were present in greater amounts under BFC. According to Alexandre [2], the oxidative metabolism of flor yeasts is an adaptive mechanism allowing cells to survive under biological aging conditions in wine.

As can be seen from Figure 2, a large number of proteins involved in glutamate biosynthesis (the chemical reactions and pathways resulting in the formation of glutamate, the anion of 2-aminopentanedioic acid) were detected under BFC, possibly because this process is related and effected by proteins corresponding to the TCA cycle.

### 2.2. Stress Damage Prevention and Repair

Mitochondrial DNA (mtDNA) can be severely damaged by the presence of reactive oxygen species produced by the respiratory chain under biological aging conditions. In fact, ROS are reported as more deleterious on mtDNA than they are on nuclear DNA, probably as a result of the greater proximity of the former to the major sites of the endogenous production of ROS [3]. In this work, we detected proteins preventing these adverse effects and various others repairing the resulting damage. Thus, four out the five proteins that regenerate NADPH in *S. cerevisiae* were detected under BFC and two under NBFC (Table 2). NADPH is required to reduce oxidized glutathione in the glutathione reductase reaction, which makes it a key factor for ROS peroxide decomposition [16]. Furthermore, proteins involved in maintaining the structure and integrity of the mitochondrial genome were found in greater contents under BFC (0.94 mol % overall) than under the reference NBFC (0.28 mol % in total). Aco1p, which plays an essential role in mtDNA maintenance, exhibited the greatest difference, with 0.35 mol % under BFC and 0 mol % under NBFC. As a component of mitochondrial nucleoids, this protein may directly protect mtDNA from cumulative point mutations and ssDNA breaks. Hsp78p, which was also detected under BFC, is known to be required for resumption of mitochondrial respiratory function [14]. Cooperatively with mitochondrial Ssc1p, more abundant under BFC, Hsp78p dissociates, resolubilizes and refolds aggregates of proteins in the mitochondrial matrix that have likely been damaged [17] by ROS [18]. The combined detrimental effects of ROS and other stresses (e.g., high acetaldehyde and ethanol concentrations) under BFC may also explain the large number of proteins involved in nucleosome disassembly detected under BFC. This biological process is mainly related to transcription, but is also associated with the responses to DNA damage stimulus and DNA break repair.

Proteins involved in the unfolding and refolding of other proteins (both with *p*-value < 0.1) were also detected in increased amounts under BFC (Figure 2). These processes are performed often when the proteins are in a non-functional or denatured state. Among these proteins, Hsp78p acts mainly in the mitochondrial matrix [18], and Hsp104p is required, in concert with other proteins (including Ydj1p, Ssa1p, Hsp26p), to refold proteins damaged by an environmental stress, like ethanol. Both Hsp78p and Hsp104p are expressed at higher levels in respiring cells than in fermenting cells [19].

### 2.3. Protein Biosynthesis

In this work, we obtained high GO Term ratios for processes, such as translational elongation, de novo protein folding and rRNA export from the nucleus, as well as for cellular components such as the small subunit of the ribosome and the translation elongation factor 1 complex.

High frequencies of more abundant proteins were found under BFC (Table 2). One protein (viz., aerobically-induced Hyp2p; [20]) was detected in much greater contents under BFC than under NBFC (1.37 mol % versus 0.08 mol %). Such a large difference may have resulted from the protein being involved in the stress response mechanism and in the maintenance of cell wall integrity, which may be especially important under BFC due to the risk of a high ethanol concentration damaging the cellular wall [21,22].

Besides, five out of six proteins assisting the folding of a nascent peptide chain into its correct tertiary structure (‘de novo’ protein folding) in *S. cerevisiae* were detected under BFC versus only two under NBFC (Table 2). Hsp82p, which was only detected under BFC, is additionally required for the refolding of denatured proteins back into their native conformations [23]; also, Hsp82p transcription is strongly induced by stress [24]. Elevated frequencies of proteins forming the ribosome subunits and translation elongation factor 1 complex were found under BFC (Table 1 and the Supplementary Material).

The facts that the total number of proteins detected was greater under NBFC (611) than under BFC (413) and that 80% of the proteome is expressed during the log phase (14) led us to expect a greater number of abundant proteins involved in translation functions under NBFC. Thus, we may hypothesize that protein biosynthesis becomes important in the oxidative environment provided by the typically high ethanol concentrations under BFC, where flor yeasts are active in replacing proteins damaged by ROS and/or a high ethanol content of the medium [21,22,25]. This hypothesis is consistent with previous results of Zara et al. [26], who found a 4.3-fold higher frequency of translation genes overexpressed in biofilm cells compared to non-biofilm cells.

### 2.4. Cell Wall and Extracellular Region

Cell wall is a structure that affords protection from stresses and contributes to cell morphogenesis. The more abundant proteins under BFC annotated in the fungal-type cell wall showed a *p*-value lower than 0.1. Elevated frequencies of more abundant proteins and specifically cell wall proteins contributing to stress responses were reported under BFC; for instance, Ecm33p, which is specifically involved in the response to oxidative stress and ethanol [27,28], and Ssa2p and Gas1p, which are also involved in oxidative stress resistance mechanisms [29].

Besides the previous functions, cell wall proteins have been associated with the velum-forming ability of flor yeasts. According to some authors, the presence of molecules such as β-glucans or mannoproteins, which increase the hydrophobicity of cell surfaces, affects such ability [5,30,31,32]. Furthermore, specific cell wall proteins such as the GPI-anchored cell surface glycoprotein Flo11p play a crucial role in the biofilm formation process [6,7,32,33,34]. In this study, the proteome analysis did not detect Flo11p even though several proteins involved in Ras protein signal transduction, which mediates its transcription [35], were reported in greater amounts under BFC. Ten cell wall proteins were also anchored membrane components, and six of them (Ccw14p, Crh1p, Ecm33p, Gas1p, YJL171C and YNL190W) were more abundant under BFC; and so were others involved in the synthesis of β-glucans under NBFC (Skn1p, Gsc2p and kre6p). Furthermore, ten of the twenty-three cell wall proteins identified were mannoproteins, namely Ccw14p, Crh1p, Ecm33p, Gas1p and Ynl190wp, which were more abundant under BFC; Cwp1p, Gas3p, Gas5p and Hsp150p, the last of which is extensively mannosylated, which prevailed under NBFC; and Pst1p, which was also extensively mannosylated, but found in similar amounts in BFC and NBFC. Based on the phenotype analyses for null mutants, the cell wall proteins Tdh1p, Ccw14p and Ssa2p, the first two being more concentrated under BFC, but the last being present in similar amounts under both conditions, had a favorable effect on biofilm formation; by contrast, Cwp1p and Tdh2p, which were more concentrated under NBFC and BFC, respectively, had an adverse effect on it.

As can be seen from the Supplementary Material, some proteins exhibited large differences between BFC and NBFC. This was especially the case of Bgl2p, followed by Scw10p and Ecm33p. The glucanase Bgl2p was only detected under BFC (1.01 mol %); this is the most important protein in the cell wall, which is involved in its maintenance and incorporation of newly synthesized mannoprotein molecules [36,37]. This cell wall protein may be involved in β-glucan degradation to α-glucose and function biosynthetically as a trans-glycosylase resulting in (1→3)-β-d-glucan chain elongation and intrachain 1,6-β linkages into 1,3-β glucan; the latter contributes cell wall structural rigidity [36,38]. Ecm33p, which was detected at 0.63 mol % under BFC, but absent under NBFC, is an extensively *N*-glycosylated GPI-anchored protein also contributing to cell wall integrity and correct assembly of the mannoprotein outer layer of cell walls. Scw10p and its paralogue Scw4p, which was present under both BFC and NBFC, albeit in markedly different amounts (>2-fold under BFC), are two glycosylated glucanases. Scw4p synthesis has been associated with increased adhesion [39] and hence with biofilm consistency. However, Cappellaro et al. [40] found the *scw4 scw10* double mutant to exhibit defects in mating. Hence, high contents of both proteins under BFC may indicate that flor yeasts are undergoing sexual reproduction.

Nineteen cell wall proteins were also detected in the extracellular region, which contained several others not present in cell walls (viz., Acb1p, Pst2p, Suc2p, Uth1p, Ygp1p and YOR389Wp). Suc2p, Ygp1p and Yor389wp were present in greater amounts under BFC than under NBFC; the former two at up to 0.97 mol % and 1.12 mol %, respectively. Suc2p catalyzes the hydrolysis of molecules such as fructan, raffinose, inulin and sucrose, none of which were present in the studied media. Its unexpectedly increased contents under BFC may have resulted from the yeasts responding to a medium containing no fermentable carbon sources. By contrast, Ygp1p exhibited the largest differences between BFC and NBFC. Ygp1p is an extensively *N*-glycosylated cell wall-related secretory protein potentially involved in cellular adaptations prior to the stationary phase, cellular amino acid metabolism, cell wall assembly and, also, possibly, biofilm formation [41]. Destruelle et al. [42] observed *YGP1* induction in response to nutrient limitation and repression by high glucose concentrations. Based on the large differences in contents between BFC and NBFC, its functions and the fact that until now it has only been located in the extracellular region [43], Ygp1p may be a constituent of the extracellular matrix, the composition of which remains unknown [44]. A high content in protein Pst2p, but similar under BFC (0.55 mol %) and NBFC (0.42 mol %) here, was previously correlated with the formation of a yeast biofilm [41].

### 2.5. Peroxisome

Vandenbosch et al. [41] performed a screening study of the *S. cerevisiae* deletion mutant bank and suggested that the peroxisome also contributes to biofilm formation. In this work, we detected six different peroxisomal proteins under BFC, all of which were more abundant than under NBFC (Table 1). Pex1p, which was detected under BFC only, was the sole peroxisomal protein related to biofilm formation. A total of ten proteins in this class have been linked to this phenotype in *S. cerevisiae*. Pex1p is a component of the peroxisomal protein import machinery positively regulated by proteins involved in the depression of glucose-repressible genes. Null mutants for this protein show phenotypes as decreased biofilm formation [41] and decreased tolerance to ethanol [28,45]. Two other peroxisomal proteins (Hyr1p and Pnc1p) have been associated with hydrogen peroxide resistance; both were present in greater amounts under BFC. Hyr1p functions as a sensor and transducer of hydroperoxide stress; thus, it responds to hydroperoxide stress by oxidizing (triggering) the transcription activator *YAP1*, which triggers the transcription of genes involved in the oxidative stress response pathway. This protein, which may also play a direct role in hydroperoxide scavenging, is the most active in peroxide and lipid hydroperoxide reduction among three closely related peroxiredoxins in *S. cerevisiae* (viz., Gpx1p, Gpx2p and Hyp1p/Hyr1p, none of which were detected here). Finally, Pnc1p catalyzes nicotinamide de-amidation, an early step in the NAD^+^ salvage pathway potentially related to the response to oxidative stress. Two other proteins that were only detected under BFC (Cit2p and Idp3p) participate in the glyoxylate cycle, which is essential for growth on two-carbon compounds. Cit2p also participates in the TCA cycle. On the other hand, Idp3p may act in the production of NADPH for fatty acid and sterol synthesis, which is important for flor yeast flotability [46]. Pox1p, which was only found under NBFC, participates in fatty acid metabolism.

### 2.6. Vacuoles, Proteolysis Regulation and Apoptosis

The vacuolar proteins detected under BFC were highly expressed (particularly those in the luminal portion). The combined contents in vacuolar proteins were up to 10-times higher under BFC than under NBFC. Three of the eleven proteins detected in *S. cerevisiae* vacuolar lumina were present under both conditions (Table 1). Two such proteins (Npc2p and Pep4p) were found in increased amounts under BFC, but did not exceed 0.16 mol % under NBFC. Thus, Npc2p was present at 0.92 mol % under BFC, but absent under NBFC; similarly, Pep4p was present at 1.20 mol % under BFC, but only 0.16 mol % under NBFC. The vacuole plays both lytic and storage functions. The large difference in Npc2p content between BFC and NBFC may have resulted from the protein being involved in biofilm development. In fact, this protein catalyzes the intermembrane transfer of phosphatidylglycerol and phosphatidylinositol, the glycosylated forms of which act as anchoring molecules for cell wall mannoproteins, such as Flo11p [47,48]. On the other hand, Pep4p is an aspartyl protease (proteinase A) involved in protein turnover after oxidative damage, as well as in the post-translational regulation of *S. cerevisiae* vacuole proteinases, including itself, plasma membrane transporters and the alkaline phosphatase Pho8p (not detected here).

As can be seen from Figure 2, three of the four proteins involved in the regulation of proteolysis and, more specifically, in the downregulation of endopeptidase activity in *S. cerevisiae* were detected under BFC. In response to hydrogen peroxide, Pep4p migrates outside into the cytoplasm to mediate mitochondrial and nucleoporin degradation [49,50]. Proteinase A is also required for processes, such as chronological aging, apoptosis and the yeast retrograde response pathway, which is important for gene expression changes during mitochondrial dysfunction. Apoptosis has been suggested as an altruistic response to severe oxidative damage in various microorganisms [51,52]. Further, Lloyd et al. [53] hypothesized that unrepaired cellular constituents and components (especially mitochondria) eventually lead to cellular senescence and apoptosis after a finite number of respiratory cycles. Regulated apoptosis can prevent the release of toxic cellular components, and also, it can provide nutrients for healthy cells. For these reasons, flor yeasts can be assumed to undergo apoptosis during biological aging of wine. In this work, eight of the twenty-five apoptosis-related proteins in *S. cerevisiae* were detected under BFC, with seven in greater contents than under NBFC (*p*-value < 0.001); on the other hand, six proteins were detected under NBFC, three in greater amounts than under BFC (see Table 2). Proteins involved in the downregulation of apoptosis were also detected in relatively large numbers. Thus, two of the six proteins in this class present in *S. cerevisiae* (viz., Bmh1p and Stm1p) were detected under both BFC and NBFC and a third (Bmh2p) under BFC only (see Table 2).

### 2.7. Catabolism of Several Sugars

As can be seen in Table 2, three of the four arabinose and d-xylose catabolic proteins present in *S. cerevisiae* (viz., Gcy1p, Gre3p and Ypr1p) were detected under BFC, but only one (Ypr1p) was under NBFC in a lower content than under BFC. Since neither sugar was present in the studied media, these proteins may be involved in a different process. In fact, all are somehow associated with oxidative stress response and adaptation [54]. Thus, Gre3p and Ypr1p production is induced by oxidative stress [55,56], whereas Gcy1p expression is downregulated by glucose [57]. Alternatively, the yeasts may have responded to the lack of glucose in the medium by synthesizing these proteins to avoid starvation.

### 2.8. Amino Acid Metabolism

A number of proteins involved in glycine, isoleucine and arginine metabolism were detected under BFC. Four such proteins participate in glycine metabolism in *S. cerevisiae*; three were more abundant under BFC (*p*-value < 0.05), and so was the other (Gcv2p) under NBFC (Table 2). It has to be also considered that the glycine cleavage complex, which catalyzes the reversible oxidation of glycine, showed a *p*-value lower than 0.1 (Figure 2 and also highlighted in Figure 3). Glycine is the smallest of the twenty amino acids commonly found in proteins and a glucogenic amino acid by virtue of its ability to be converted into serine by serine hydroxymethyltransferase; in turn, serine can be converted back into the glycolytic intermediate 3-phosphoglycerate or to pyruvate by serine/threonine dehydratase. Two of the proteins detected under BFC (Shm1p and Shm2p) contribute to the interconversion of serine and glycine. Both are involved in the production of precursors for purine, pyrimidine, amino acid and lipid biosynthesis [58]. Furthermore, they contribute to tetrahydrofolate interconversion and possess a high GO Term ratio (Figure 2); tetrahydrofolate is an essential cofactor for the transfer of one-carbon units from donor molecules into important biosynthetic pathways leading to methionine (pathway ratio of 4.44), purine and pyrimidine. Three of the four proteins related to glycine catabolism were only detected under BFC (Gcv1p, Gcv3p, Lpd1p) and only one under NBFC, Gcv2p (Table 2). Gcv3p, which is a component of the glycine decarboxylase complex (GDC), was detected at up to 1.11 mol %. GDC is a multi-enzyme complex catalyzing the reversible oxidative cleavage of glycine into CO_2_ and NH_3_ and connecting the metabolism of one-, two- and three-carbon compounds [59,60,61]. The other subunits of the GDC complex are Gcv1p, Lpd1p and Gcv2p; the first two were only detected under BFC and so was the third under NBFC.

Biologically-synthesized isoleucine can be produced as two different isomers, namely: an L isomer, which is one of the twenty-two proteinogenic amino acids (i.e., the building blocks of proteins) and a D isomer, which acts as a carbon, nitrogen and energy source. A total of ten proteins related to isoleucine synthesis in *S. cerevisiae* were identified; five (Bat1p, Hom2p, Hom6p, Ilv3p and Mmf1p) were present in greater amounts under BFC; three (Bat2p, Ilv2p and Ilv6p) were found in higher contents under NBFC; and the other (Ilv5p) was detected in similar amounts under both conditions. Bat1p, with 0.47 mol % under BFC and 0.09 mol % under NBFC, is known to be highly expressed during the logarithmic phase of growth and downregulated during the stationary phase. Mmf1p, which was detected in increased amounts under BFC (0.52 mol %), is associated with mitochondrial translation and to mitochondrial DNA maintenance. On the other hand, the isoleucine degradation process was also highlighted (Figure 3): four proteins more abundant under BFC (Adh2p, Bat1p, Sfa1p and Thi3p), while one under NBFC (Bat2p). The same proteins also play a role in the leucine degradation pathway, which showed an equal value of pathway ratio (Table 3 and Figure 3). Another degradation pathway was highlighted, which is the catabolism of valine. This is one of the few amino acids that *S. cerevisiae* can use as a carbon source and the degradation of which results in the fusel alcohols, important flavor and aroma compounds [62].

### 2.9. Glycolysis/Gluconeogenesis

A total of twenty-six proteins was annotated to the glycolysis/gluconeogenesis process in *S. cerevisiae*; fifteen were detected under BFC and fourteen under NBFC (Table 2). Ratios above two were also reported for glycolysis and glucose fermentation pathways (Table 3). Ten of the fifteen glycolytic proteins detected under BFC were also involved in the gluconeogenetic process required for yeast growth on ethanol or glycerol as the carbon source [10]. This may account for the large number of proteins found under BFC. Two of the five glycolytic proteins detected in greater contents under BFC than under NBFC (viz., Tdh1p and Tdh2p) participate in gluconeogenesis and so does one exhibiting the opposite trend (viz., Pgi1p).

### 2.10. Folate Biosynthesis

As for the folate, or vitamin B9, six proteins were found more abundant under BFC related to the synthesis of this compound (Ade3p, Gcv1p, Gcv3p, Lpd1p, Shm1p and Shm2p). Ade3p, Shm1p and Shm2p specifically play a role in the folate polyglutamylation. This compound contains healthy properties and is used as a food additive because humans are unable to synthesize it [63,64]. Due to this interesting property, future metabolic analyzes should be focused on detecting the presence and regulation of this compound in Sherry wines.

### 2.11. Cellular Components, Biological Processes and Pathways of Relevance under NBFC

Most of the proteins located in organelles or involved in biological process/pathways associated with cell division and gene transcription were detected in increased amounts or even exclusively under NBFC (see Figure 1, Figure 2 and Figure 3). The thioredoxin and glutathione/glutaredoxin systems help maintain the reduced environment of the cell and play a significant role in defending the cell against oxidative stress, which can happen at the initial phase of the fermentation when oxygen is rapidly consumed (Figure 4) and ROS can be released. They also have been proposed to play a role in the biosynthesis of purines and pyrimidines as required for DNA synthesis, protein folding and regulation and sulfur metabolism [65,66]. On the other hand, histidine is relevant, as its biosynthesis is linked to the pathways of nucleotide formation.

**Figure 6:**
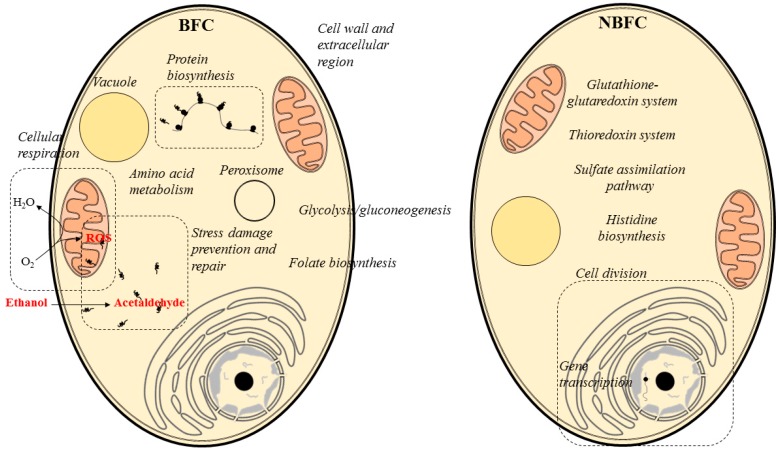
Yeast biological processes and organelles highlighted by the abundance of proteins detected under BFC and NBFC.

After compiling all data, we speculate that *S. cerevisiae* flor yeast at the early stages of biofilm formation exerts oxidative metabolism and produces a protein machinery that prevents and protects the yeast from reactive oxygen species (ROS) derived from respiration (Figure 6). Further, the presence of proteins involved in the bioconversion of the carbon source (ethanol) to other compounds, such as polysaccharides, amino acids or folate, were reported. Cell wall and extracellular region proteins, both related to the flor biofilm formation and maintenance, were found abundant under BFC. On the other hand, proteins associated with cell division and gene transcription prevailed under NBFC, indicating that the yeast reproduction is highly active at this point (Figure 6).

The cellular components, biological processes and pathways examined may be essential for flor yeasts to survive under BFC, such as those prevailing in biologically-aged wines. However, genetic experiments are needed to confirm the need for these yeasts to synthesize the target proteins. This knowledge may be useful to plan effective technical strategies for the genetic improvement of flor yeast strains with a view toward extending their survival during biological aging of Sherry wines and improving their production yield as a result. Thus, shortening aging times under a flor velum to avoid the excessive production of acetaldehyde can help preserve the olfactory profile of the wine and obtain safe wines. Furthermore, a deeper insight into the role of proteins in flor yeasts may be highly useful with a view toward advancing cell biofilm-related immobilization technology.

## 3. Materials and Methods

Data treated in the present manuscript were extracted from Moreno-García et al. [10], where the *Saccharomyces cerevisiae* flor yeast strain, inoculum and cultivation conditions are described. In order to follow dissolved oxygen content in the medium, the Oxymeter device CRIMSON OXI 92 was used. Samples were subjected to serial dilution and the total concentration of cells counted on a Z2 Coulter particle counter and analyzer (Beckman). Previously, samples were passed on 0.01 M of an EDTA solution and then counted in order to avoid cell flocculation. Cell viability was determined by spreading 100-µL volumes of diluted suspension onto YPD agar plates and counting colonies after 48 h at 28 °C.

Methods for the collection of the cells and protein extraction, fractionation, identification and quantification are indicated in Moreno-García et al. [4,10].

An explanation for the overall proteome was sought by performing a GO Term and pathway search for the most abundant proteins (specifically, those present in greater amounts under BFC or NBFC and those detected only under either condition), using the SGD YeastMine tool and UniProt databases [67,68]. Proteins were classified into “cellular component” (viz., part of a cell or its extracellular environment in which a gene product is located) and “biological process” (a collection of molecular events with a well-defined beginning and end), both using gene ontology classification; and into “pathways” using YeastMine. The tool “GO Term finder” from SGD was used to determine the *p*-value for each annotation; this is the probability or chance of seeing at least “x” number of genes out of the total “n” genes in the list annotated to a particular GO term, given the proportion of genes in the whole genome that are annotated to that GO Term. GO Terms with *p*-values lower than 0.1 have been distinguished through the manuscript. Further, it should be noted that some proteins may be present in more than one cell compartment or exhibit more than one molecular function; and involved in more than one process as a result. Moreover, the false discovery rate (FDR) has been also determined through the GO Term finder. This is obtained by making 50 simulated runs with aleatory genes and counting the average number of times a *p*-value as good as or better than a *p*-value generated from the real data is seen.

The discussion focuses on the GO Terms or pathways leading to the greatest numbers of more abundant proteins (including specific proteins and common proteins with more content in one of the conditions) gathered under BFC or NBFC relative to the other condition and is based on the GO Term or pathway ratio, which was calculated as follows:

(1) $$\text{GO Term or pathway ratio}=\frac{(\text{BFC more abundant proteins in a GO Term or pathway}/\text{BFC total proteins})\times 100}{(\text{NBFC more abundant proteins in a GO Term or pathway}/\text{NBFC total proteins})\times 100}$$

The GO Terms and pathways with a ratio higher than 2 were assumed to play an important role under BFC and the one’s with ratio lower than 1/2 to be important under NBFC. By contrast, those GO Terms gathering only 1–2 proteins under either condition against 0–1 under the other were excluded as they might have led to erroneous conclusions.

## Figures and Tables

**Table 1:** Proteins ^1^ detected in relevant cellular components under BFC and NBFC.

Cellular Component	BFC Only	BFC and NBFC	NBFC Only
Small ribosomal subunit	Mrp4p, Rps0Bp, Rps15p	Rps0Ap, **Rps5p**	
Translation elongation factor 1 complex	Tef4p	**Efb1p**, **Tef1p**	
Respiratory chain	Cor1p, Cyb2p	**Qcr6p**	
Anchored component of membrane	Crh1p, Dcw1p, Ecm33p, Plb1p, YJL171C, YNL190W	**Ccw14p**, Cwp1p, **Gas1p**, Pst1p	Gas3p, Gas5p
Fungal-type cell wall	Bgl2p, Crh1p, Ecm33p, Pho5p, Scw10p, Tos1p, YJL171C, YNL190W	**Ccw14p**, Cwp1p, Exg1p, **Gas1p**, Pst1p, **Scw4p**, Ssa1p, Ssa2p, **Tdh1p**, **Tdh2p**, Tdh3p	Gas3p, Gas5p, Hsp150p, Scw11p
Glycine cleavage complex	Gcv1p, Gcv3p, Lpd1p		Gcv2p
Extracellular region	Bgl2p, Crh1p, Ecm33p, Pho5p, Scw10p, Suc2p, Tos1p, Ygp1p, YNL190W, YOR389W	Acb1p, **Ccw14p**, Cwp1p, Exg1p, Gas1p, Pst1p, Pst2p, **Scw4p**, Ssa1p, Ssa2p	Gas3p, Gas5p, Hsp150p, Scw11p, Uth1p
Peroxisome	Cit2p, Hyr1p, Idp3p, Pex1p	**Aat2p**, **Pnc1p**	Gpd1p, Pex19p, Pox1p
Fungal-type vacuole lumen	Npc2p, Tfs1p	**Pep4p**	Cps1p, Prb1p
DNA replication pre-initiation complex			Mcm7p, Psf1p, Sld5p
DNA-directed RNA polymerase III complex			Rpc19p, Rpc37p, Rpo26p
Chromosome, telomeric region		Def1p	Gbp2p, Rap1p, Rfa3p, Rrm3p, Sub2p,
Replication fork protection complex			Ctf4p, Mcm7p, Psf1p, Sld5p, Spt16p

^1^ Proteins detected under both conditions, but in at least twice greater amounts under BFC are shown in bold, and those with higher content under NBFC are underlined.

**Table 2:** Proteins ^1^ involved in relevant biological processes under BFC and NBFC.

Biological Processes	BFC Only	BFC and NBFC	NBFC Only
ATP synthesis coupled proton transport	Atp14p, Atp16p, Atp1p, Atp5p	Atp2p	
Arabinose catabolic process	Gcy1p, Gre3p	**Ypr1p**	
‘De novo’ protein folding	Hsp82p, Rot1p, Ydj1p	Hsc82p, Hsp60p	
D-xylose catabolic process	Gcy1p, Gre3p	**Ypr1p**	
Hydrogen ion transmembrane transport	Nha1p, Pma1p	Cox4p, Cox6p, **Qcr6p**	
Negative regulation of apoptotic process	Bmh2p	**Bmh1p**, **Stm1p**	
Negative regulation of endopeptidase activity	Pbi2p, Rfu1p, Tfs1p		
Protein unfolding	Hsp104p, Hsp78p	**Ssc1p**	
Ras protein signal transduction	Bmh2p, Srv2p	**Bmh1p**	
rRNA export from nucleus	Rps0Bp, Rps15p	Rps0Ap, **Rps5p**	
Tricarboxylic acid cycle	Aco1p, Cit1p, Cit2p, Fum1p, Idh1p, Idh2p, Idp3p, Kgd1p, Kgd2p, Mdh1p, Sdh1p	**Lsc1p**	Aco2p
Glutamate biosynthetic process	Cit1p, Cit2p, Idh1p, Idh2p, Put2p		Gdh1p
Protein refolding	Hsp78p, Hsp82p, Ydj1p	Hsc82p, **Hsp10p**, Hsp60p, Ssa1p, **Ssc1p**, Sse1p	Mge1p
Glycine catabolic process	Gcv1p, Gcv3p, Lpd1p		Gcv2p
Glycine metabolic process	Gcv1p, Shm1p	**Shm2p**	Gcv2p
Tetrahydrofolate interconversion	Shm1p	**Ade3p**, **Shm2p**	Met13p
Apoptotic process	Bir1p, Kex1p, Mca1p, Pet9p, Por1p	**Cpr3p**, **Tdh2p**, Tdh3p	Esp1p, Oye2p, Ymr074cp
Glycolytic process	Glk1p, Hxk1p, YLR446W	**Cdc19p**, Eno1p, Eno2p, Fba1p, Gpm1p, Pdb1p, Pgi1p, Pgk1p, **Tdh1p**, **Tdh2p**, Tdh3p, Tpi1p	Hxk2p, Pfk1p
Nucleosome disassembly	Asf1pAsf1p, Npl6p, Rsc4p	**Rsc8p**	Nap1p, Rsc2p
Isoleucine biosynthetic process	Hom2p, Ilv3p, Mmf1p	**Bat1p**, **Hom6p**, Ilv5p	Bat2p, Ilv2p, Ilv6p
Arginine biosynthetic process	Yer069wp	**Arg1p**, **Arg7p**, Cpa2p	Arg4p, Arg8p
Mitochondrial genome maintenance	Aco1p, Hsp78p, Kgd2p	Ilv5p	Rpo41p, Rrm3p
NADPH regeneration	Ald4p, Idp3p, YMR315W	Ald6p	Zwf1p
Translational elongation	Tef4p, Tuf1p	**Efb1p**, Eft1p, Hyp2p, Rpp0p, Rpp2Ap, Rpp2Bp, Rps21Ap, **Stm1p**, **Tef1p**	Rpp1Bp, Yef3p,
DNA-dependent DNA replication			Abf1p, Ctf4p, Dpb4p, Psf1p, Sld5p, Spt16p, Yil082w-Ap
Regulation of transcription from RNA polymerase II promoter			Dst1p, Fcp1p, Iki3p, Med4p, Met32p, Ssn2p, Std1p, Ylr278cp
DNA replication		Pol30p	Abf1p, Ctf4p, Dpb4p, Mcm7p, Pol12p, Psf1p, Rfa3p, Rnr4p, Rrm3p, Sgs1p, Sld5p, Spt16p

^1^ Proteins detected under both conditions, but in at least twice greater amounts under BFC are shown in bold, and those with higher content under NBFC are underlined.

**Table 3:** Proteins ^1^ involved in relevant pathways under BFC and NBFC.

Pathway	BFC Only	BFC and NBFC	NBFC Only
2-Ketoglutarate dehydrogenase complex	Kgd1p, Kgd2p, Lpd1p		
Aerobic respiration, electron transport chain	Cor1p, Sdh1p	Cox4p, Cox6p, **Qcr6p**	
Superpathway of TCA cycle and glyoxylate cycle	Aco1p, Cit1p, Cit2p, Fum1p, Idh1p, Idh2p, Kgd1p, Kgd2p, Mdh1p, Sdh1p	**Lsc1p**	Pyc2p
**Pathway**	**BFC Only**	**BFC and NBFC**	**NBFC Only**
Isoleucine degradation	Adh2p, Thi3p	Adh1p, **Bat1p**, Pdc1p, Pdc5p, **Sfa1p**	Bat2p
Leucine degradation	Adh2p, Thi3p	Adh1p, **Bat1p**, **Sfa1p**	Bat2p
Folate biosynthesis	Gcv1p, Gcv3p, Lpd1p, Shm1p	**Ade3p**, **Shm2p**	Gcv2p, Met13p
Folate polyglutamylation	Shm1p	**Ade3p**, **Shm2p**	Met13p
Glycine cleavage complex	Gcv1p, Gcv3p, Lpd1p		Gcv2p
Superpathway of methionine biosynthesis	Hom2p, Met2p	**Hom6p**, Met17p, Met6p	
Valine degradation	Adh2p	Adh1p, **Bat1p**, Pdc1p, Pdc5p, **Sfa1p**	Bat2p
Glucose fermentation	Adh2p, Ald4p, Glk1p, Hxk1p	Adh1p, Ald6p, **Cdc19p**, Eno1p, Eno2p, Fba1p, Gpm1p, Pdc1p, Pdc5p, Pgi1p, Pgk1p, **Tdh1p**, **Tdh2p**, Tdh3p, Tpi1p	Hxk2p, Pfk1p,
Pathways of chorismate	Aro2p, Shm1p	**Ade3p**, **Aro4p**, **Shm2p**, Trp5p	Aro3p, Aro8p, Trp2p
Arginine biosynthesis	Yer069wp	**Arg1p**, **Arg7p**, Cpa2p	Arg4p, Arg8p
Glycolysis		**Cdc19p**, Eno1p, Eno2p, Fba1p, Gpm1p, Pgi1p, Pgk1p, **Tdh1p**, **Tdh2p**, Tdh3p, Tpi1p	Pfk1p
Superpathway of serine and glycine biosynthesis	Agx1p, Shm1p	Ser1p, **Shm2p**	Ser33p
Superpathway of threonine and methionine biosynthesis	Hom2p, Met2p	**Hom6p**, Met17p, Met6p	Thr4p
Superpathway of threonine biosynthesis	Hom2p	**Aat2p**, **Hom6p**	Pyc2p, Thr4p
Glutathione-glutaredoxin system		Grx1p, Grx2p	Glr1p, Grx3p, Grx4p, Grx5p
Thioredoxin system		Trx1p, Trx2p	Trr1p
Sulfate assimilation pathway		Ecm17p	Met10p, Met14p
Histidine biosynthesis			His3p, His4p, His7p

^1^ Proteins detected under both conditions, but in at least twice greater amounts under BFC are shown in bold, and those with higher content under NBFC are underlined.

## Supplementary Materials

Supplementary materials can be found at www.mdpi.com/1422-0067/18/4/720/s1.

## Abbreviations

ROS	Reactive oxygen species
BFC	Biofilm formation condition
NBFC	Non-biofilm formation condition
SGD	*Saccharomyces* Genome Database
GO	Gene ontology
TCA	Tricarboxylic acid cycle
GDC	Glycine decarboxylase complex
YPD	Yeast extract, peptone, dextrose medium
FDR	False discovery rate
